# 4-(Diphenyl­phosphino­yl)benzoic acid

**DOI:** 10.1107/S1600536808031449

**Published:** 2008-10-04

**Authors:** Yuan-Jing Li, Rong-Chang Zhang

**Affiliations:** a Faculty of Chemistry and Biology, Beihua University, Jilin City 132013, People’s Republic of China

## Abstract

Mol­ecules of the title compound, C_19_H_15_O_3_P, are connected by O—H⋯O hydrogen bonds between the carboxylic acid OH group and the phosphinoyl O atom, forming chains running along the crystallographic *b* axis.

## Related literature

For general background, see, see: Al-Farhan (1992 ▶). For related structures, see: Etter (1990 ▶); Fuquen & Lechat (1992 ▶).
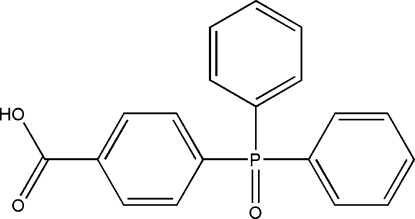


## Experimental

### 

#### Crystal data

C_19_H_15_O_3_P
*M* *_r_* = 322.28
Monoclinic, 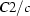

*a* = 18.018 (3) Å
*b* = 10.0921 (18) Å
*c* = 18.028 (4) Å
β = 91.467 (4)°
*V* = 3277.1 (11) Å^3^
*Z* = 8
Mo *K*α radiation
μ = 0.18 mm^−1^
*T* = 293 (2) K
0.24 × 0.21 × 0.17 mm

#### Data collection

Bruker APEX CCD area-detector diffractometer
Absorption correction: multi-scan (*SAINT*; Bruker, 1998 ▶) *T*_min_ = 0.956, *T*_max_ = 0.971
8975 measured reflections
3228 independent reflections
1796 reflections with *I* > 2σ(*I*)
*R*_int_ = 0.079

#### Refinement

*R*[*F* ^2^ > 2σ(*F* ^2^)] = 0.056
*wR*(*F* ^2^) = 0.110
*S* = 0.91
3228 reflections
208 parameters
H-atom parameters constrained
Δρ_max_ = 0.38 e Å^−3^
Δρ_min_ = −0.25 e Å^−3^

### 

Data collection: *SMART* (Bruker, 1998 ▶); cell refinement: *SAINT* (Bruker, 1998 ▶); data reduction: *SAINT*; program(s) used to solve structure: *SHELXS97* (Sheldrick, 2008 ▶); program(s) used to refine structure: *SHELXL97* (Sheldrick, 2008 ▶); molecular graphics: *SHELXTL* (Sheldrick, 2008 ▶); software used to prepare material for publication: *SHELXTL*.

## Supplementary Material

Crystal structure: contains datablocks global, I. DOI: 10.1107/S1600536808031449/bt2800sup1.cif

Structure factors: contains datablocks I. DOI: 10.1107/S1600536808031449/bt2800Isup2.hkl

Additional supplementary materials:  crystallographic information; 3D view; checkCIF report

## Figures and Tables

**Table 1:** Hydrogen-bond geometry (Å, °)

*D*—H⋯*A*	*D*—H	H⋯*A*	*D*⋯*A*	*D*—H⋯*A*
O3—H3⋯O1^i^	0.82	1.78	2.579 (3)	163

Symmetry code: (i) 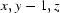.

**Fig. 1.**
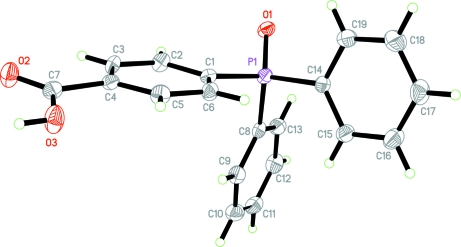
The structure of the title compound, showing the atomic numbering scheme. Displacement ellipsoids are drawn at the 30% probability level.

**Fig. 2.**
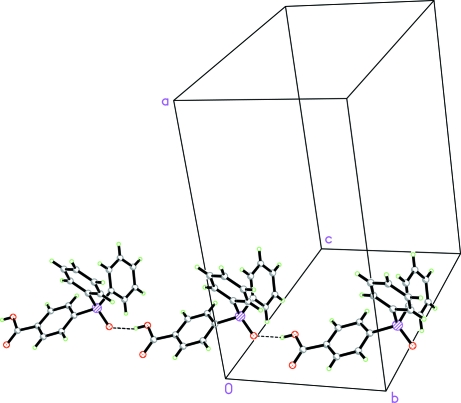
View of the chain structure of the title compound; hydrogen-bonds are drawn as dashed lines.

## supplementary crystallographic information

### Comment

Triphenylphosphine is an important intermediate in organic chemistry. So far, its derivative, triphenylphosphine P-oxide with diverse hydrogen-bond donors, have been extensively studied (Al-Farhan, 1992). However, 4-(triphenylphosphine oxide)formic acid, as an important derivative of triphenylphosphine has been rarely studied (Fuquen & Lechat, 1992). The title compound was synthesized from 4-(diphenylphosphino)benzoic acid.

The O—H···O hydrogen bonds between the O atoms of the oxide group and the carboxylate group link the molecules to chains running along the crystallographic b axis.

### Experimental

4-(Diphenylphosphino)benzoic acid (5 mmol) and hydrogen peroxide (0.5 ml) were dissolved in a mixture of CH_3_CH_2_OH and water solution (40 ml) (CH_3_CH_2_OH: water = 3:1). The mixture was refluxed for 1 h, after cooling, this mixture was diluted with water, immediately resulting in a white precipitate, which was washed with water. Crystals of the title compound were obtained by recrystallization from CH_3_CH_2_OH.

### Refinement

All H atoms  were positioned geometrically (O—H = 0.82 Å, C—H = 0.93 Å) and refined as riding, with *U*_iso_(H) = 1.2*U*_eq_(C,O).

### Crystal data

**Table:**

C_19_H_15_O_3_P	*F*(000) = 1344
*M**_r_* = 322.28	*D*_x_ = 1.306 Mg m^−^^3^
Monoclinic, *C*2/*c*	Mo *K*α radiation, λ = 0.71073 Å
Hall symbol: -C 2yc	Cell parameters from 3228 reflections
*a* = 18.018 (3) Å	θ = 1.1–26.0°
*b* = 10.0921 (18) Å	µ = 0.18 mm^−^^1^
*c* = 18.028 (4) Å	*T* = 293 K
β = 91.467 (4)°	Block, white
*V* = 3277.1 (11) Å^3^	0.24 × 0.21 × 0.17 mm
*Z* = 8	

### Data collection

**Table:**

Bruker APEX CCD area-detector diffractometer	3228 independent reflections
Radiation source: fine-focus sealed tube	1796 reflections with *I* > 2σ(*I*)
graphite	*R*_int_ = 0.079
φ and ω scans	θ_max_ = 26.0°, θ_min_ = 2.3°
Absorption correction: multi-scan (SAINT; Bruker, 1998)	*h* = −22→16
*T*_min_ = 0.956, *T*_max_ = 0.971	*k* = −11→12
8975 measured reflections	*l* = −21→22

### Refinement

**Table:**

Refinement on *F*^2^	Primary atom site location: structure-invariant direct methods
Least-squares matrix: full	Secondary atom site location: difference Fourier map
*R*[*F*^2^ > 2σ(*F*^2^)] = 0.056	Hydrogen site location: inferred from neighbouring sites
*wR*(*F*^2^) = 0.110	H-atom parameters constrained
*S* = 0.91	*w* = 1/[σ^2^(*F*_o_^2^) + (0.0279*P*)^2^] where *P* = (*F*_o_^2^ + 2*F*_c_^2^)/3
3228 reflections	(Δ/σ)_max_ < 0.001
208 parameters	Δρ_max_ = 0.38 e Å^−^^3^
0 restraints	Δρ_min_ = −0.25 e Å^−^^3^

### Special details

**Table:**

Geometry. All e.s.d.'s (except the e.s.d. in the dihedral angle between two l.s. planes) are estimated using the full covariance matrix. The cell e.s.d.'s are taken into account individually in the estimation of e.s.d.'s in distances, angles and torsion angles; correlations between e.s.d.'s in cell parameters are only used when they are defined by crystal symmetry. An approximate (isotropic) treatment of cell e.s.d.'s is used for estimating e.s.d.'s involving l.s. planes.
Refinement. Refinement of *F*^2^ against ALL reflections. The weighted *R*-factor *wR* and goodness of fit *S* are based on *F*^2^, conventional *R*-factors *R* are based on *F*, with *F* set to zero for negative *F*^2^. The threshold expression of *F*^2^ > σ(*F*^2^) is used only for calculating *R*-factors(gt) *etc*. and is not relevant to the choice of reflections for refinement. *R*-factors based on *F*^2^ are statistically about twice as large as those based on *F*, and *R*- factors based on ALL data will be even larger.

### Fractional atomic coordinates and isotropic or equivalent isotropic displacement parameters (Å^2^)

**Table:**

	*x*	*y*	*z*	*U*_iso_*/*U*_eq_	
C1	0.10622 (15)	−0.1711 (3)	0.17928 (17)	0.0272 (7)	
C2	0.07579 (16)	−0.2100 (3)	0.11102 (17)	0.0346 (8)	
H2	0.0682	−0.1471	0.0738	0.041*	
C3	0.05660 (16)	−0.3408 (3)	0.09760 (17)	0.0332 (8)	
H3A	0.0367	−0.3658	0.0516	0.040*	
C4	0.06720 (16)	−0.4344 (3)	0.15324 (17)	0.0283 (8)	
C5	0.09413 (17)	−0.3952 (3)	0.22220 (17)	0.0353 (9)	
H5	0.0994	−0.4572	0.2601	0.042*	
C6	0.11340 (16)	−0.2644 (3)	0.23532 (17)	0.0330 (8)	
H6	0.1313	−0.2390	0.2820	0.040*	
C7	0.05236 (17)	−0.5773 (3)	0.13561 (19)	0.0342 (8)	
C8	0.22186 (16)	0.0143 (3)	0.14358 (16)	0.0304 (8)	
C9	0.27202 (18)	−0.0893 (3)	0.13654 (18)	0.0401 (9)	
H9	0.2615	−0.1718	0.1566	0.048*	
C10	0.33799 (19)	−0.0698 (4)	0.0995 (2)	0.0466 (10)	
H10	0.3713	−0.1396	0.0951	0.056*	
C11	0.35436 (19)	0.0512 (4)	0.06954 (18)	0.0453 (10)	
H11	0.3986	0.0633	0.0449	0.054*	
C12	0.30524 (19)	0.1546 (4)	0.07603 (19)	0.0463 (10)	
H12	0.3161	0.2369	0.0558	0.056*	
C13	0.23927 (18)	0.1359 (3)	0.11296 (18)	0.0395 (9)	
H13	0.2063	0.2062	0.1172	0.047*	
C14	0.15618 (17)	0.0219 (3)	0.28792 (17)	0.0307 (8)	
C15	0.22564 (17)	0.0031 (3)	0.32047 (18)	0.0384 (8)	
H15	0.2650	−0.0225	0.2914	0.046*	
C16	0.2371 (2)	0.0220 (3)	0.3956 (2)	0.0473 (10)	
H16	0.2843	0.0106	0.4168	0.057*	
C17	0.1788 (2)	0.0578 (3)	0.4395 (2)	0.0516 (10)	
H17	0.1864	0.0706	0.4902	0.062*	
C18	0.1090 (2)	0.0743 (4)	0.4076 (2)	0.0597 (11)	
H18	0.0692	0.0969	0.4370	0.072*	
C19	0.09794 (19)	0.0576 (3)	0.3324 (2)	0.0483 (10)	
H19	0.0509	0.0704	0.3113	0.058*	
O1	0.07883 (11)	0.09304 (18)	0.16115 (11)	0.0348 (6)	
O2	0.02276 (13)	−0.6151 (2)	0.07916 (13)	0.0469 (7)	
O3	0.07785 (13)	−0.6563 (2)	0.18890 (13)	0.0527 (7)	
H3	0.0690	−0.7336	0.1777	0.079*	
P1	0.13600 (5)	−0.00176 (8)	0.19091 (5)	0.0298 (2)	

### Atomic displacement parameters (Å^2^)

**Table:**

	*U*^11^	*U*^22^	*U*^33^	*U*^12^	*U*^13^	*U*^23^
C1	0.0234 (17)	0.0297 (18)	0.0285 (19)	0.0009 (14)	−0.0005 (14)	0.0021 (15)
C2	0.040 (2)	0.0334 (19)	0.030 (2)	−0.0041 (17)	−0.0055 (16)	0.0100 (16)
C3	0.033 (2)	0.037 (2)	0.029 (2)	−0.0061 (17)	−0.0061 (15)	−0.0037 (16)
C4	0.0237 (18)	0.0276 (18)	0.033 (2)	−0.0006 (15)	−0.0004 (15)	0.0024 (15)
C5	0.041 (2)	0.0318 (19)	0.032 (2)	0.0044 (17)	−0.0061 (16)	0.0088 (16)
C6	0.036 (2)	0.034 (2)	0.028 (2)	0.0016 (17)	−0.0059 (15)	−0.0018 (16)
C7	0.029 (2)	0.038 (2)	0.035 (2)	−0.0020 (17)	0.0025 (16)	0.0019 (18)
C8	0.0320 (19)	0.0346 (19)	0.0243 (18)	−0.0038 (17)	−0.0057 (14)	−0.0023 (16)
C9	0.041 (2)	0.039 (2)	0.040 (2)	−0.0025 (18)	−0.0021 (18)	−0.0019 (17)
C10	0.037 (2)	0.059 (3)	0.044 (2)	0.001 (2)	0.0037 (18)	−0.005 (2)
C11	0.034 (2)	0.070 (3)	0.032 (2)	−0.008 (2)	0.0045 (16)	−0.003 (2)
C12	0.050 (2)	0.048 (2)	0.041 (2)	−0.014 (2)	0.0019 (19)	0.0049 (19)
C13	0.037 (2)	0.042 (2)	0.039 (2)	−0.0014 (18)	−0.0004 (17)	−0.0004 (18)
C14	0.0297 (19)	0.0278 (19)	0.034 (2)	−0.0020 (15)	−0.0006 (15)	−0.0029 (15)
C15	0.0323 (19)	0.048 (2)	0.035 (2)	−0.0013 (18)	0.0005 (15)	−0.0020 (19)
C16	0.041 (2)	0.060 (3)	0.041 (2)	−0.003 (2)	−0.0068 (18)	0.000 (2)
C17	0.062 (3)	0.065 (3)	0.028 (2)	−0.005 (2)	0.0014 (19)	−0.0037 (19)
C18	0.049 (3)	0.091 (3)	0.039 (2)	0.008 (2)	0.0107 (19)	−0.013 (2)
C19	0.035 (2)	0.068 (3)	0.042 (2)	0.006 (2)	−0.0018 (18)	−0.009 (2)
O1	0.0290 (12)	0.0307 (12)	0.0442 (15)	0.0002 (11)	−0.0080 (11)	0.0026 (11)
O2	0.0594 (17)	0.0369 (14)	0.0436 (16)	−0.0040 (12)	−0.0152 (13)	−0.0018 (12)
O3	0.0748 (18)	0.0269 (13)	0.0553 (18)	−0.0046 (13)	−0.0226 (14)	0.0037 (12)
P1	0.0285 (5)	0.0294 (5)	0.0314 (5)	−0.0020 (4)	−0.0029 (4)	−0.0002 (4)

### Geometric parameters (Å, °)

**Table:**

C1—C6	1.384 (4)	C10—H10	0.9300
C1—C2	1.391 (4)	C11—C12	1.375 (4)
C1—P1	1.802 (3)	C11—H11	0.9300
C2—C3	1.384 (4)	C12—C13	1.390 (4)
C2—H2	0.9300	C12—H12	0.9300
C3—C4	1.388 (4)	C13—H13	0.9300
C3—H3A	0.9300	C14—C15	1.382 (4)
C4—C5	1.381 (4)	C14—C19	1.385 (4)
C4—C7	1.499 (4)	C14—P1	1.793 (3)
C5—C6	1.384 (4)	C15—C16	1.379 (4)
C5—H5	0.9300	C15—H15	0.9300
C6—H6	0.9300	C16—C17	1.380 (5)
C7—O2	1.199 (3)	C16—H16	0.9300
C7—O3	1.322 (3)	C17—C18	1.380 (4)
C8—C13	1.385 (4)	C17—H17	0.9300
C8—C9	1.389 (4)	C18—C19	1.374 (5)
C8—P1	1.793 (3)	C18—H18	0.9300
C9—C10	1.392 (4)	C19—H19	0.9300
C9—H9	0.9300	O1—P1	1.4952 (19)
C10—C11	1.371 (4)	O3—H3	0.8200
			
C6—C1—C2	118.8 (3)	C12—C11—H11	120.1
C6—C1—P1	122.5 (2)	C11—C12—C13	119.8 (3)
C2—C1—P1	118.7 (2)	C11—C12—H12	120.1
C3—C2—C1	121.0 (3)	C13—C12—H12	120.1
C3—C2—H2	119.5	C8—C13—C12	121.1 (3)
C1—C2—H2	119.5	C8—C13—H13	119.4
C2—C3—C4	119.6 (3)	C12—C13—H13	119.4
C2—C3—H3A	120.2	C15—C14—C19	118.8 (3)
C4—C3—H3A	120.2	C15—C14—P1	123.7 (3)
C5—C4—C3	119.6 (3)	C19—C14—P1	117.5 (2)
C5—C4—C7	121.6 (3)	C16—C15—C14	120.7 (3)
C3—C4—C7	118.8 (3)	C16—C15—H15	119.7
C4—C5—C6	120.5 (3)	C14—C15—H15	119.7
C4—C5—H5	119.7	C15—C16—C17	120.1 (3)
C6—C5—H5	119.7	C15—C16—H16	120.0
C5—C6—C1	120.4 (3)	C17—C16—H16	120.0
C5—C6—H6	119.8	C18—C17—C16	119.6 (3)
C1—C6—H6	119.8	C18—C17—H17	120.2
O2—C7—O3	124.3 (3)	C16—C17—H17	120.2
O2—C7—C4	124.0 (3)	C19—C18—C17	120.2 (4)
O3—C7—C4	111.7 (3)	C19—C18—H18	119.9
C13—C8—C9	118.4 (3)	C17—C18—H18	119.9
C13—C8—P1	118.5 (3)	C18—C19—C14	120.7 (3)
C9—C8—P1	123.1 (3)	C18—C19—H19	119.6
C8—C9—C10	120.2 (3)	C14—C19—H19	119.6
C8—C9—H9	119.9	C7—O3—H3	109.5
C10—C9—H9	119.9	O1—P1—C8	111.49 (14)
C11—C10—C9	120.6 (3)	O1—P1—C14	112.67 (13)
C11—C10—H10	119.7	C8—P1—C14	107.23 (14)
C9—C10—H10	119.7	O1—P1—C1	111.40 (12)
C10—C11—C12	119.8 (3)	C8—P1—C1	106.75 (14)
C10—C11—H11	120.1	C14—P1—C1	106.98 (14)

### Hydrogen-bond geometry (Å, °)

**Table:**

*D*—H···*A*	*D*—H	H···*A*	*D*···*A*	*D*—H···*A*
O3—H3···O1^i^	0.82	1.78	2.579 (3)	163

Symmetry codes: (i) *x*, *y*−1, *z*.
